# Effect of the S100A9/AMPK pathway on PM2.5-mediated mouse lung injury

**DOI:** 10.22038/ijbms.2024.80242.17374

**Published:** 2025

**Authors:** Yunxia Li, Yuxin Bai, Shiyu Tang, Ye Sun, Zhe Wang, Biao Yang, Guangyan Liu

**Affiliations:** 1 Department of Respiratory and Critical Care Medicine, The Fourth People’s Hospital of Shenyang, Shenyang 110000, China; 2 Department of Pathogen Biology, Shenyang Medical College, Shenyang. No. 146, Huanghe North Street, Shenyang, People’s Republic of China; 3 Department of Pathophysiology, Shenyang Medical College, Shenyang. No. 146, Huanghe North Street, Shenyang, China; 4 Department of Medical Oncology, Affiliated Zhongshan Hospital of Dalian University, Dalian, People’s Republic of China; 5 The Key Laboratory of Biomarker High Throughput Screening and Target Translation of Breast and Gastrointestinal Tumor, Dalian University, Dalian, People’s Republic of China; 6 Graduate school, Shenyang Medical College, Shenyang. No. 146, Huanghe North Street, Shenyang, People’s Republic of China

**Keywords:** AMPK ATP, Lung injury, PM2.5, ROS, S100A9

## Abstract

**Objective(s):**

Particulate matter 2.5 (PM2.5), particles with an aerodynamic diameter less than 2.5 µm, affect lung function and increase respiratory disease incidence and mortality rate. The molecular mechanism of lung injury and epithelial damage after PM2.5 exposure is not completely clear.

**Materials and Methods:**

Mouth-nose exposure of mice was performed with PM2.5 or neutral saline. *In vitro* experiments were conducted to investigate the role of the S100A9/AMPK pathway in PM2.5-mediated lung injury.

**Results:**

PM2.5 exposure in mice caused lung epithelial damage, alveolar wall thickening, and alveolar wall structure destruction. The 16S rRNA sequencing results suggested that the microecology structure of lung tissue was altered after PM2.5 exposure. Proteomic sequencing was performed to explore the underlying mechanism, and 71 differentially expressed proteins were identified. KEGG database analysis of the up-regulated differential proteins revealed regulatory networks, including fat digestion and absorption, the AMPK signaling pathway, and the PPAR signaling pathway. Moreover, PM2.5 exposure in mice increased the level of S100A9 and ROS, leading to reduction of the ATP level. To achieve a sufficient energy supply by increasing fatty acid transfer and oxidation, activated AMPK up-regulates CD36 and CPT1, which leads to mitochondrial damage of PM2.5-exposed cells and injury or death of lung epithelial cells. siRNA-S100A9 and AMPK inhibitors significantly reduced the occurrence of cell damage.

**Conclusion:**

These results may help to clarify biomarkers and specific mechanisms of lung tissue injury induced by PM2.5 exposure.

## Introduction

Air pollution particles are composed of a heterogeneous mixture of components, and particulate matter (PM) 2.5 (PM with an aerodynamic diameter of less than 2.5 µm) is considered the main air pollutant. PM2.5 is composed of metal components and organic substances. Epidemiological investigations and experimental studies have demonstrated that PM2.5 can cause lung function damage and increase the incidence and mortality of respiratory and cardiovascular diseases (1-5). The effect of PM2.5 on lung parenchyma is related to its heterogeneity, and the components of inhalable PM may have varying sources in different geographical locations. Therefore, it is necessary to research PM2.5 in other regions to reduce the negative impact of air pollution on health. Thus, we researched the toxic mechanism of PM2.5 in Shenyang, China.

PM2.5 is a harmful, inhalable mixture that damages human health through a strong cumulative effect, carrying many harmful substances (6). In our previous study, we collected PM2.5 samples from Shenyang city and performed source apportionment. The complex components of PM2.5 were identified and included metal ions, acid ions, and polycyclic aromatic hydrocarbons (7). An association between PM2.5 exposure and the change in the local microenvironment has been discovered in many pulmonary diseases, including chronic obstructive pulmonary disease and acute lung injury (8, 9). 

PM2.5 is easy to inhale into the respiratory system, leading to deposition in the bronchi and lung alveoli, and it induces lung tissue damage as a result of oxidative stress, airway local immunosuppression, partial exfoliation of epithelial cells, and exudation and infiltration of inflammatory cells in airways (10). PM2.5-induced lung injury has been associated with the secretion of numerous cytokines and chemokines and the generation of cellular reactive oxygen species (ROS), which stimulate inflammatory signals and subsequent lung injury (11, 12). Previous studies reported that exposure to PM induces the production of interleukin-6 (IL-6), monocyte chemotactic protein 1 (MCP-1), metalloproteinases-8 (MMP-8), the receptor for advanced glycation end-products (RAGE), and tissue inhibitor of metalloproteinase-1 (TIMP-1) in bronchoalveolar lavage fluid (BALF) (13, 14). Oxidative stress, a critical imbalance between the production of ROS and antioxidants, leads to severe damage to lung tissue (15). PM2.5 can stimulate alveolar macrophages and alveolar epithelial cells to produce ROS. Excessive ROS can cause damage to airway epithelial and alveolar cells, inducing DNA damage, airway epithelial damage, and airway inflammation. The inflammatory reaction and ROS play a role in cell damage or death (16-18).

Increasing studies have provided evidence for the relationship between PM2.5 and human health, mainly including the effects of cytotoxicity and reactive oxygen species (19-21). Oxidative stress has been proposed to be an important molecular mechanism of PM2.5-induced lung epithelial injury. It plays an important role in lung tissue remodeling in PM-induced human diseases (22). Mitochondria, important cellular organelles, are involved in multiple biological processes and serve as a powerhouse for cell energy production. Mitochondrial health is critical to lung tissue and associated with lung epithelial survival and fitness, including initiation and amplification of cell damage. The increasing recognition of mitochondrial etiology in a range of human diseases has led to the emergence of research on mitochondrial toxicants and the role of mitochondria in PM2.5-induced lung injury (23, 24). However, the molecular mechanism of lung tissue injury and epithelial damage caused by PM2.5 exposure is not completely clear. 

Our research explores the role of PM2.5 in lung epithelial injury in Shenyang to prevent and reduce lung tissue injury and improve human health. We conducted PM2.5 exposure experiments in mice and used *in vitro* experiments to investigate the role of the S100 calcium-binding protein A9/AMP-activated protein kinase phosphorylation (S100A9/AMPK) pathway in PM2.5-mediated lung injury. These findings help clarify the underlying molecular mechanism of PM2.5-induced lung injury, providing potential therapeutic targets for lung injury and making new contributions to human health.

## Materials and Methods

***PM2.5 sample from Shenyang, China***

The concentrated PM2.5 solution (5 mg/ml in neutral saline) was collected in the center of downtown Shenyang, China, and stored at -80 ^°^C in our laboratory. The PM2.5 samples utilized in this study were obtained using previously described methods (7). 

***Animals and PM2.5 administration***

The BALB/c mice purchased from Vital River, CP (Beijing, China), were utilized at eight weeks of age (16-20 g). They were randomly divided into a PM2.5 exposure group (n=10) and a Neutral saline (NS) control group (n=10). All studies were conducted per the Animal Research: Reporting of *In Vivo* Experiments (ARRIVE) guidelines and approved by the College’s Ethics Committee. The mice received the same food and water and were kept at the same ambient conditions in the Laboratory Animal Care Center. After a 2-week acclimation period, all mice were executed by exposure to PM2.5 and neutral saline using the Mouth-nose Exposed system (Beijing Huironghe Technology Co., Ltd., China).

In the PM2.5-exposed group, the animals were exposed to concentrated PM2.5 at 750 μg/cm^3^ for 6 hr per day, five days per week for a total of 2 weeks, and the control group animals were treated with NS in the same condition. Twenty-four hours following the last exposure, mice were euthanized, and blood serum and lung tissue samples were collected for further analysis. 

***Histopathology analysis and apoptosis assay***

The lung tissues were inflated and fixed with 10% neutral formalin. Then, they were dehydrated with increasing ethanol (EtOH) concentrations and embedded in paraffin. Five-micrometer-thick paraffin sections were cut from each specimen, and hematoxylin and eosin (H&E) staining and an apoptosis assay (Elabscience Biotechnology Co., Ltd, China) were performed as instructed. 

***Transmission electron microscopy (TEM) insection***

Two apexes of the left lung and experimental cell were selected and immersed in 2.5% glutaraldehyde at 4 ^°^C. After washing with phosphate buffer solution, they were fixed with 1% osmium tetroxide, dehydrated in a graded series of ethanol, embedded in Araldite and then polymerized overnight at 37 ^°^C. We cut the ultrathin section of lung tissue with ultramicrotome (LKB-I, Sweden). Thin sections were mounted on copper grids, stained with lead citrate, and observed with the H-7650 electron microscopy (HITACHI, Japan). 

***Microbial community analysis***

After harvest, BALF from three PM2.5 exposed and three control mice were immediately flash-frozen in liquid nitrogen. According to the Illumina Hiseq 2500 Sequencing System protocols, the 16S rRNA sequencing was performed by Gene Denovo Biotechnology Company (Guangzhou, China). Microbial DNA was extracted (Omega Biotek, U.S.), and using the primers of 341F and 806R, the 16S rDNAV3-V4 region of ribosomal RNA was amplified by PCR. Venn analysis was performed in R upon identifying unique and common OTUs between groups. The Principal component analysis (PCA) was calculated and plotted in R. The abundance statistics of each taxonomy and phylogenetic tree were constructed in a Perl script. The representative sequences were classified into organisms based on the Greengenes Database (https://www.arb-silva.de/). 

***Proteomic sequencing and enrichment analysis***

The lung tissue from three PM2.5 exposed and three control mice were immediately flash frozen in liquid nitrogen after harvest. Proteomic sequencing was performed by Gene Denovo Biotechnology Company (Guangzhou, China). Protein identifications were accepted if they could achieve an FDR less than 1.0% by the Scaffold Local FDR algorithm. Proteins that contained similar peptides and could not be differentiated based on MS/MS analysis alone were grouped to satisfy the principles of parsimony. Protein quantification was carried out in those proteins identified in all the samples with unique spectra ≥2. Protein relative quantification was based on the ratios of reporter ions, which reflect the relative abundance of peptides. The Mascot search results were averaged using medians and quantified. Proteins with fold change in a comparison >1.2 or <0.83 and unadjusted significance level (*P*<0.05) were considered differentially expressed. Proteins were annotated against GO, KEGG, and COG/KOG databases to obtain their functions. Significant GO functions and pathways were examined within differentially expressed proteins with a P-value≤0.05. 

***Cell culture and treatments***

Pulmonary epithelial cells (BEAS-2B) were obtained from the China Infrastructure of Cell Line Resource, grown in RPMI-1640 medium (Hyclone, USA) with 10% fetal bovine serum (Hyclone, USA) and 1% penicillin-streptomycin (Hyclone, USA), and kept at 37 ^°^C in 5% CO_2_. The cells were seeded at a density of 1.0×10^4^ in triplicate in 96-well plates, tackled with different concentrations of PM2.5 in 0, 25, 50, 100, and 200 μg/ml, and cultured in a CO_2_ incubator for 24 hr.

We performed cell viability and detected cell damage in each group, including the PM2.5-exposed group, siRNA-S100A9, and PM2.5-exposed combined group, AMPK Inhibitor (Dorsomorphin) and PM2.5-exposed combined group, and siRNA-S100A9 and AMPK Inhibitor and PM2.5-exposed combined group. The control group included the NS exposed group, siRNA-S100A9 group, AMPK Inhibitor group, and siRNA-S100A9 and AMPK Inhibitor group.

***Cell proliferation assays***

According to the instructions, cell viability was assessed with CellTiter 96 AQueous One Solution Reagent (Promega, USA). Absorbance was taken on an infinite M200 Pro Reader (TECAN, Switzerland) with a wavelength of 490 nm. All assays were measured in triplicate experiments. 

***RNA extraction and quantitative real-time PCR***

Performing Quantitative real-time PCR (qRT-PCR) to detect the fold changes of candidate genes, we used an ABI Prism 7500 (Applied Biosystems, USA) and SYBR Master Mix (Applied Biosystems, USA) according to the manufacturer’s instruction. Total RNA was extracted and treated with DNase I and reverse transcripted (Promega, USA). Primer pairs used for QPCR are shown in Table 1. The results of the QPCR of mRNA levels were determined after normalization to β-actin expression, and the relative expression levels were calculated using the 2^-ΔΔCt^ method. 

***Measurement of MDA, ROS, and ATP***

Cell damage, including cell membrane damage, mitochondrial ROS production, and (adenosine triphosphate) ATP level, was detected. Experimental cells were determined using the Lipid Peroxidation MDA Assay kit, Reactive Oxygen Species Assay Kit, and ATP Assay Kit (Beyotime Biotechnology, China) according to the manufacturer’s instructions. 

***Statistical analysis***

The data were analyzed using GraphPad Prism 6 (GraphPad Software, CA, USA). The data were reported as the mean ± SD of three independent experiments. ANOVA was used to evaluate the differences between groups. Statistical comparisons were made using an unpaired two-tailed Student’s t-test for two groups. Differences were considered significant if *P*<0.05. 

## Results

***PM2.5 Exposure-induced lung injury and morphological changes***

**Figure 1:**
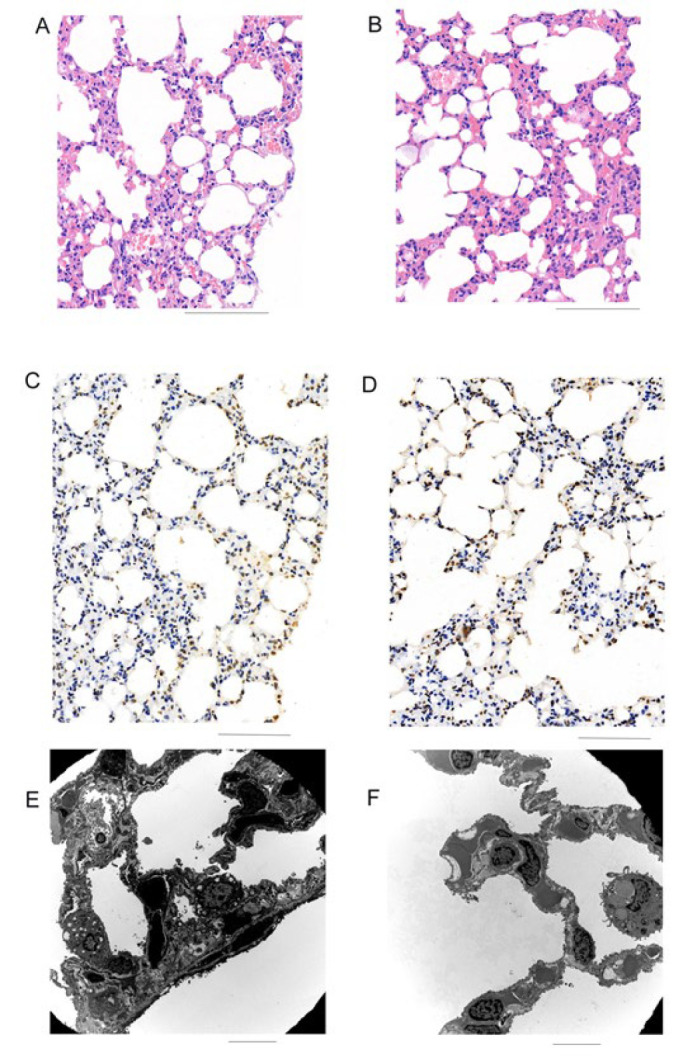
PM2.5-exposed induced lung injury

To detect the histological changes in lung tissues and the state of lung injury, we utilized a “real-world” PM2.5-exposed system to perform a mouth-nose exposure with PM2.5 or NS. Then, we carried out the H&E staining, apoptosis assay, and transmission electron microscopy. To test the morphological changes, we performed HE staining on the lung tissue samples obtained from the PM2.5-exposed and NS control groups (Figure 1A and 1B). The structural changes of alveolar sacs and alveoli were small in the NS group. However, after PM2.5 exposure, we found that the alveolar epithelium was incomplete, the alveolar structure was damaged, the gas-blood barrier was damaged, and scattered hemorrhage could be seen in the alveolar cavity. With the apoptosis assay, as shown in Figures 1C and 1D, compared with the control group, we confirmed that the apoptosis of alveolar epithelial in the PM2.5 exposure group significantly increased. According to the results of TEM (Figures 1E and 1F), we observed significant thickening of the alveolar wall and destruction of its structure following PM2.5 exposure. These results indicate that the occurrence of lung tissue injury can be induced in “real-world” PM2.5-exposed conditions.

***Profiling bacterial sequences on Lung tissue flora diversity***

**Figure 2:**
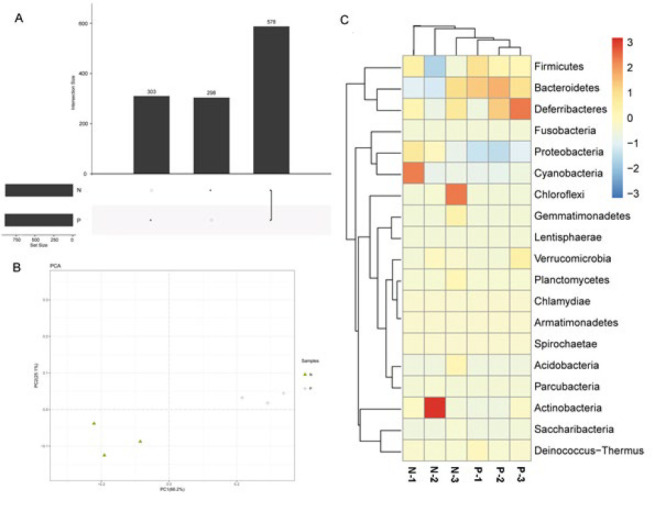
Bacterial diversity in lung tissue

Utilizing an operational taxonomic unit (OTU) method from the SILVA repositories of bacterial 16S sequences, we confirmed the 578 OTUs; that is to say, 589 bacteria have been found (Figure 2A). Wherein we found 298 OTUs in the control group and 303 OTUs in the BALF of the PM2.5-exposed group. To investigate the alterations with abundance and diversity in each group, we analyzed the Principal coordinates analysis (PCoA) and found that the flora communities in the lung tissues of PM2.5-exposed mice were distinct from the NS exposure (Figure 2B). According to the 16S rRNA sequencing data in the lung tissues, we selected the OTU taxonomic units with an abundance greater than 2%. We constructed a species classification diagram at the phylum level (Figure 2C). At the phylum level, the bacteria with the highest abundance were Firmicutes, and there was no significant difference between the PM2.5-exposed group and the NS control group. In the lung tissue exposed to PM2.5, the proportion of Bacteroides and Proteobacteria was approximately twice as high as the NS control group. The above results suggested that PM2.5 exposure caused significant changes in the flora structure of mouse lung tissue.

***Proteomic sequencing***

**Figure 3:**
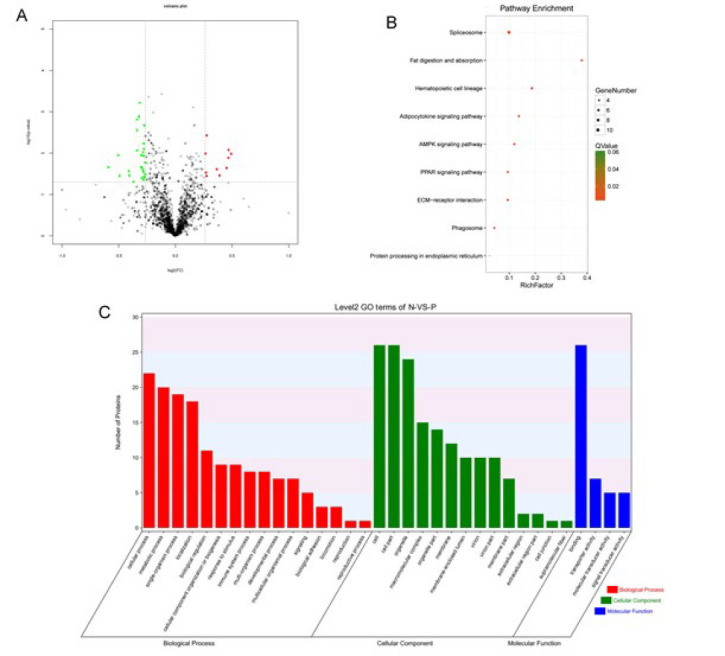
Proteomic sequencing of mouse lung tissue

In order to reveal the mechanism of PM2.5-exposed lung injury and the change of bacterial diversity in lung tissue, we carried out proteomic sequencing of mouse lung tissue. Compared with the NS control group, we found 27 up-regulated and 44 down-regulated proteins (Figure 3A). Analysis of the up-regulated target protein pathway revealed that it affects nine different types of pathways, including spliceosome, fat digestion and absorption, hematopoietic cell lineage, adipocytokine signaling pathway, AMPK signaling pathway, PPAR signaling pathway, ECM-receptor interaction, phagosome, and protein processing in the endoplasmic reticulum (Figure 3B). As shown in Figure 3C, under GO analysis, we found the functions targeted by these proteins were responses to biological processes, molecular functions, and cellular components. At the same time, the distribution of differential proteins was clarified in cellular function. Proteomic sequencing provides direction for subsequent pathway analysis and key protein exploration.

***PM2.5 exposure induced the BEAS-2B damage***

**Figure 4:**
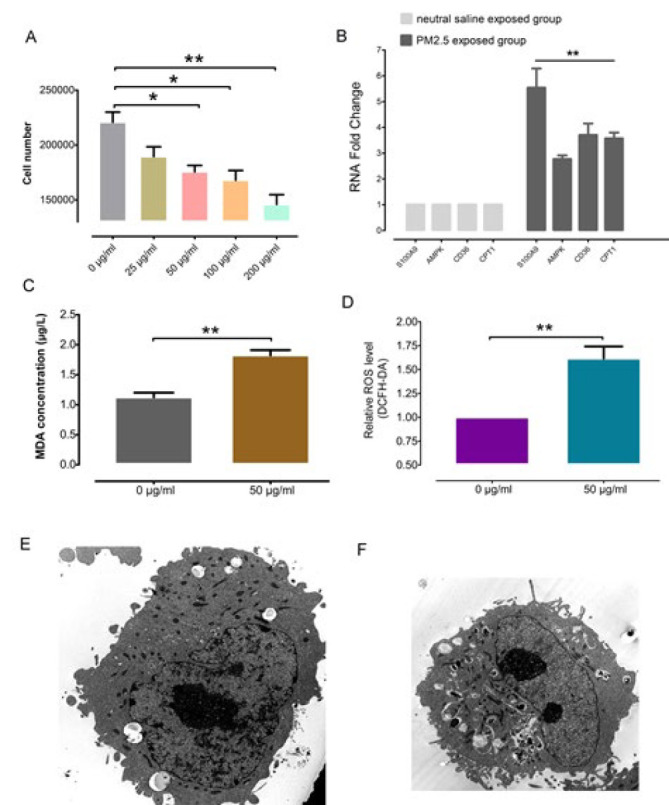
PM2.5 exposure induced BEAS-2B cell damage

To determine the concentration of PM2.5, a cell proliferation experiment was conducted with different PM2.5 concentrations of 0, 25, 50, 100, and 200 μg/ml. Absorbance was defined at a wavelength of 490 nm after PM2.5 exposure. As shown in Figure 4A, we found that 50 μg/ml PM2.5 statistically began to inhibit cell proliferation significantly. Therefore, we used 50 μg/ml PM2.5 exposure concentration. In order to verify the tendency of differential protein expression, we carried out the QPCR experiment (Figure 4B). We confirmed that the expression trend of S100A9, AMPK, CD36, and CPT1 was consistent with the proteomic sequencing in mice. At the same time, we tested the lipid oxidation level with MDA (Figure 4C). Compared with the NS control group, the level of MDA in the PM2.5-exposed group was significantly higher, indicating that PM2.5 exposure induced cell membrane damage. Next, since ROS can oxidize non-fluorescent DCFH to produce fluorescent DCF, we utilized the DCFH-DA probe to assess the cellular ROS level (Figure 4D). The results confirmed that PM2.5 exposure increased ROS and oxidative stress levels in BEAS-2B cells. According to the TEM results (Figures 4E and F), after the PM2.5 exposure, we observed black PM2.5 particles in the cytoplasm, concentrated chromatin at the edge of the nuclear membrane, and a significant increase in lipid droplets around the mitochondria. These results showed that PM2.5 exposure induced BEAS-2B cell damage.

***Effect of S100A9/AMPK pathway on PM2.5-mediated BEAS-2B damage***

**Figure 5:**
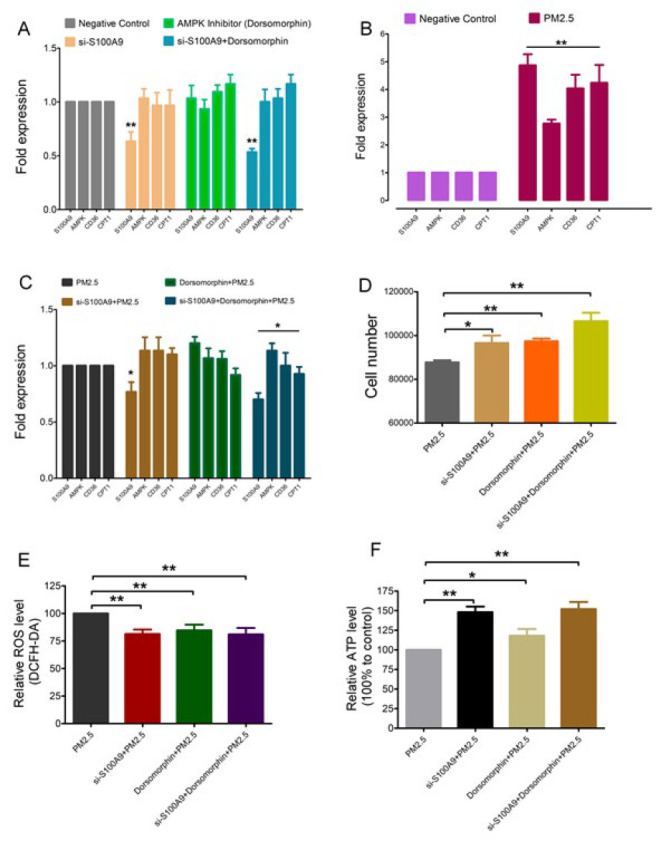
S100A9/AMPK signaling pathway in PM2.5-induced cell damage

To determine the role of the S100A9/AMPK signaling pathway in PM2.5-induced cell damage, we exposed BEAS-2B cells to a 50 μg/ml concentration of PM2.5. In addition, we treated the cells with siRNA-S100A9 interference and Dorsomorphin, an AMPK inhibitor, at a concentration of 5 mM/ml. We observed that exposure to PM2.5 increased the transcription level of S100A9. Additionally, the siRNA-S100A9 treatment led to a significant decrease in the transcription level of S100A9, as indicated in Figures 5A and B. However, compared with the PM2.5-exposed group, in the siRNA-S100A9 group, Dorsomorphin combined with the PM2.5-exposed group, the level of AMPK increased, but to some degree, the level of CD36 and CPT1 began to decrease (Figure 5C). The results of the cell proliferation assay (Figure 5D) show that the use of siRNA-S100A9 combined with Dorsomorphin could significantly increase the number of cell proliferation and that the application of two regulatory factors could lessen the damage of PM2.5-exposed cells. At the same time, utilizing DCFH-DA, we analyzed the ROS level of cells (Figure 5E). siRNA-S100A9, Dorsomorphin or the siRNA-S100A9 combined with Dorsomorphin could significantly reduce the ROS level following PM2.5 exposure. On the other hand, under the same treatment conditions, we analyzed the ATP level of cells in each group. As depicted in Figure 5F, either the individual manipulation of siRNA-S100A9 or Dorsomorphin could enhance the ATP levels of PM2.5-exposed cells. However, the group treated with siRNA-S100A9 combined with Dorsomorphin showed the highest ATP level. These results demonstrate that the S100A9/AMPK signal pathway is involved in PM2.5-induced cell damage.

**Table 1:** List of forward primers and reverse primers for qRT-PCR

Gene	Forward Primer	Reverse Primer
S100A9	5′-CCTTCCACCAATACTCTGTGAA-3′	5′-GGTCCTCCATGATGTGTTCTAT-3′
CD36	5′-CTGTTATGGGGCTATAGGGATC-3′	5′-ACTCCATCTGCAGTATTGTTGT-3′
AMPK	5'-CAACTATCGATCTTGCCAAAGG-3'	5'-AACAGGAGAAGAGTCAAGTGAG-3'
CPT1	5′-GATTTCCATTCCTTCCCATTCG-3′	5′-CTCGTATGTGAGGCAAAACTTG-3′
β-actin	5′-CCTGGCACCCAGCACAAT-3′	5′-GGGCCGGACTCGTCATAC-3′

A and B indicate H&E staining. A. PM2.5-exposed group. Scale bar, 100 μm. B. NS-exposed control group. Scale bar, 100 μm. C and D indicate the apoptosis assay. C. Apoptosis condition in PM2.5-exposed mice. Scale bar, 100 μm. D. Apoptosis condition in neutral saline-exposed mice. Scale bar, 100 μm. E and F indicate TEM. E. Ultrastructure of PM2.5-exposed group. Scale bar, 10 μm. F. Ultrastructure of NS-exposed control group. Scale bar, 10 μm

A. Venn diagrams. B. Principal component analysis (PCA) plot of the operational taxonomic units. C. Distribution of lung tissue OTUs. N indicates lung tissue OTUs in the NS-exposed group, n=3. P indicates lung tissue OTUs in the PM2.5-exposed group, n=3

A. Volcano plot. B. Pathway analysis maps for up-regulated expressed genes. C. Annotated functional analysis of significantly up-regulated expressed genes

A. Effects of PM2.5 exposure on the proliferation of BEAS-2B (n=6, *P<0.05). B. Fold change of target genes (n=6, *P<0.05). C. Levels of MDA (n=6, *P<0.05). D. Relative ROS level (n=6, *P<0.05). E. Transmission electron microscopy inspection in NS-exposed group (n=3). Scale bar indicates 10 μm. F. Transmission electron microscopy inspection in PM2.5-exposed group (n=3). Scale bar indicates 10 μm

A. Expression of four key pathway genes (n=6, *P<0.05). siRNA-S100A9 could down-regulate the level of S100A9. B. Expression of four key pathway genes (n=6, *P<0.05). PM2.5 could up-regulate the levels of the four key pathway genes. C. Expression of four key pathway genes (n=6, *P<0.05). siRNA-S100A9 and Dorsomorphin could reverse the situation after PM2.5 exposure. D. Cell proliferation after PM2.5 exposure. E. Level of ROS after PM2.5 exposure. F. Level of ATP after PM2.5 exposure

**Figure 6:**
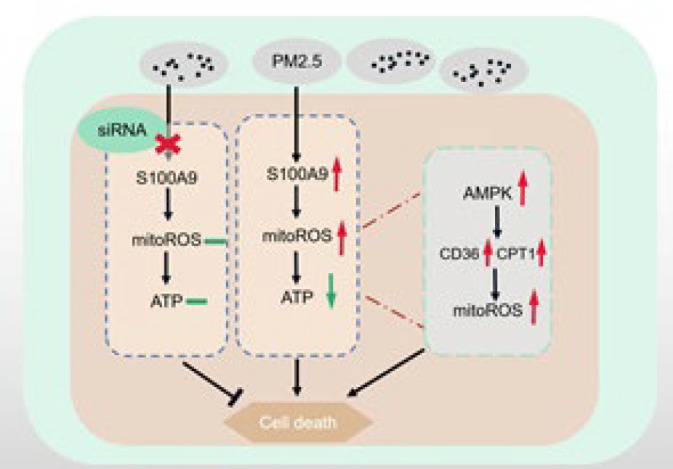
Mechanism of S100A9/AMPK pathway on PM2.5-mediated lung tissue injury

The mechanism map shows that PM2.5 can up-regulate S100A9 expression, motivate the S100A9/AMPK pathway, regulate the change of ATP levels, and induce cell damage. When siRNA knocks down the expression of S100A9, it cannot regulate ATP levels through the S100A9/AMPK pathway. Therefore, this process would not alter the production of ROS and reduce the occurrence of cell damage. The black arrow indicates the direction of the pathway; the red arrow indicates up-regulation; the green arrows indicate down-regulation; the red X represents knock-down expression; and a green horizontal line indicates no change

## Discussion

Fine particulate matter (PM2.5), the primary pollutant, has been a recent research focus. When PM2.5 enters the respiratory system, it has potentially toxic effects (25, 26). In our previous studies, we demonstrated that PM2.5 exposure causes partial exfoliation of epithelial cells and exudation and infiltration of inflammatory cells in airways; these findings indicate that PM2.5 exposure changes the local microenvironment of the lung and induces lung tissue injury (13, 14). To determine the mechanism of PM2.5-exposed lung tissue injury, *in vitro* and *in vivo* experiments were performed using PM2.5- and NS-exposed mice and cells. In mice with PM2.5 exposure, the alveolar epithelium was incomplete, scattered hemorrhage was seen in the alveolar cavity, the alveolar wall was thickened, the structure was destroyed, and the gas-blood barrier was damaged. Additionally, 16S rRNA sequencing revealed that the microecology of lung tissue changed after PM2.5 exposure. We explored the regulation of mitochondrial damage by the S100A9/AMPK pathway in lung injury after PM2.5 exposure. These results will help identify therapeutic targets and have important significance for the clinical treatment of respiratory diseases.

Respiratory tract flora is the key factor for maintaining respiratory health and plays an important role in respiratory physiology and immune homeostasis (27-29). Micro-inhaled upper respiratory tract secretions can bring oral and pharyngeal microbial communities into the lung tissue, stimulating the defense mechanism, effectively eliminating microorganisms, and blocking infection (30, 31). If the respiratory tract flora undergoes abnormal changes, it may eventually lead to local or systemic bacterial infection (32). In this study, 16S rRNA sequencing was used to objectively reduce the flora structure of lung tissue colonization. The proportion of Bacteroidetes was approximately two-fold higher in the PM2.5-exposed lung tissue compared with the NS lung tissue. However, the proportions of Bacteroidetes and cyanobacteria in the PM2.5-exposed group were only half that in the control group. These results suggest that the changes in these three major bacterial groups are concomitant with lung tissue injury after PM2.5 exposure. We analyzed the genetic diversity and abundance of the flora to explore the change in respiratory tract microecology after PM2.5 exposure and identify the dominant bacteria and changes in bacteria groups. These results lay the foundation for exploring respiratory tract bacteria that may be useful for treating lung injury. 

When PM2.5, which includes organic matter, free radicals, and metal ions, enters the respiratory system, it can stimulate cells to produce oxygen free radicals. ROS is produced during normal physiological activities; however, excessive ROS levels induce damage to the respiratory system (33). PM2.5 exposure activates the Ras homolog gene family member A/Rho-associated coiled-coil kinase (RhoA/ROCK)-dependent nuclear factor kappa-B (NF-κB) signal pathway, which enhances inflammation and lung epithelial cell damage (34). PM2.5 exposure also induces ROS and activates the interleukin 6/protein kinase B/signal transducer and activator of transcription 3/peroxisome proliferator-activated receptor gamma (IL-6/Akt/STAT3/PPARγ) signal pathway to up-regulate the level of intercellular adhesion molecule 1 (ICAM-1), thus promoting the adhesion of monocytes (35). Neutrophils can pass through the stroma and enter the protein-rich edema fluid, subsequently releasing pro-inflammatory and pro-apoptotic mediators to destroy adjacent cells, causing inflammatory damage and leading to the degradation of the base membrane and increased permeability of the alveolar-capillary barrier. 

In the present study, we found that the alveolar epithelium was incomplete, the alveolar wall was significantly thickened, and the lung parenchyma structure and the gas-blood barrier were damaged in mice after PM2.5 exposure. We performed proteomics sequencing to explore the mechanism underlying these changes and identified 71 differentially expressed proteins. KEGG analysis of the up-regulated differential proteins revealed several important regulatory pathways, including spliceosome, fat digestion and absorption, hematopoietic cell lineage, adipocytokine signaling pathway, AMPK signaling pathway, PPAR signaling pathway, ECM-receptor interaction, phagosome, and protein processing in the endoplasmic reticulum. Our findings suggest that PM2.5 exposure resulted in changes in these identified proteins, which mediate physiological mechanisms by regulating the functions of these pathways.

S100A9 is a member of the calcium-binding protein S100 protein family. S100A9 is involved in various pathophysiological activities, such as the occurrence of nerve impulses, regulation of enzyme activity, cell apoptosis, and necrosis, and it is also closely related to inflammation and tumors (36). In immune disease or inflammatory conditions, the S100A9 level increases sharply, enhancing integrin expression and recruiting monocytes to the inflammatory position (37). In epithelial cells, S100A8/A9 enhances the activity of nicotinamide adenine dinucleotide phosphate (NADPH) oxidase, strengthening ROS and NF-κB activity (38). The NF-κB pathway regulates inflammatory mediators, activates the AMPK signal, and up-regulates expression of inflammatory factors, such as tumor necrosis factor (TNF-α), IL-6, inducible nitric oxide synthase (iNOS), and cyclooxygenase 2 (COX2), subsequently inducing local inflammatory injury (39). In this study, we found that PM2.5 exposure increased the level of S100A9 and up-regulated the ROS level of exposed cells. Knockdown of S100A9 by siRNA repressed cell damage, indicating that S100A9 increases the level of ROS after PM2.5 exposure. 

AMPK is a serine-threonine kinase with a low mutation rate that plays an important role in maintaining the stability and balance of biological energy (40). AMPK is inactive under normal physiological conditions and activated by exogenous factors or when the internal environment is disturbed. When the level of ATP decreases, or newly generated ATP cannot supply the consumption of tissues and organs without delay, the dissociated AMP or ADP level increases, immediately inducing AMPK activation (41). In this study, we found that PM2.5 exposure reduced the level of ATP, leading to AMPK protein activation to reverse the low level of ATP in PM2.5-exposed cells. Activated AMPK phosphorylates downstream signal molecules and further regulates various metabolic pathways, including the metabolism of protein, fat, cholesterol, and sugar (42), to maintain a balanced and stable state in the body.

CD36 is a single-chain glycoprotein on the cell surface, belonging to the class B scavenger receptor, expressed on the surface of endothelial cells, macrophages, and other cells (43). When mice or BEAS-2B cells were exposed to PM2.5, excessive ROS was induced, along with up-regulation of PPAR signaling pathway proteins and increased expression of CD36 in cells. CD36 has a wide range of ligands *in vivo*. It regulates various physiological and pathological processes. This includes promoting the uptake of specific lipid molecules, adhesion to negatively charged biological macromolecules, and triggering inflammation, bacteriophage, and endocytosis through intracellular signal transduction (44, 45). Our study found that PM2.5 exposure increased ROS and down-regulated the ATP level of cells. To survive, PM2.5-exposed cells must increase their ATP level. Researchers have confirmed that cells can take advantage of the fat-digestion and absorption pathway to increase the intake of fatty acids and reverse the low level of ATP caused by PM2.5 exposure.

Carnitine palmitoyltransferase 1 (CPT1) is an important rate-limiting enzyme in the fatty acid β-oxidation pathway and a key factor in the balance of fatty acid metabolism (46-48). To accelerate the degradation of fatty acids and provide energy, CPT1 is activated; it directly binds long-chain fatty acids and transports them from the cytoplasm to the mitochondrial matrix for fatty acid β-oxidation. Through *in vitro* experiments, we found that siRNA-S100A9 or dorsomorphin improved the ATP level in PM2.5-exposed cells. The ATP level was the highest in cells with siRNA-S100A9 combined with dorsomorphin. Our results suggest that the S100A9/AMPK/CPT1 signaling pathway is involved in PM2.5-mediated cell damage.

To further confirm the significance of our results, we briefly analyzed the mechanism, as shown in Figure 6. In PM2.5-exposed mice or BEAS-2B cells, the expression of S100A9 is up-regulated, the level of mitoROS is increased, and ATP is reduced. With the decrease of intracellular ATP level, AMPK is activated, resulting in up-regulated expression of the downstream pathway protein CD36 and mitochondrial energy transport protein CPT1 and increased fatty acid transfer and oxidation to achieve energy supply. This leads to aggravated mitochondrial damage, resulting in injury or death of lung epithelial cells, damage of the alveolar wall tissue structure, and development of local lung tissue inflammation. These events can cause increased local capillary permeability, pulmonary edema, hemorrhage, infiltration of inflammatory cells, damage of the blood-gas barrier, pulmonary hypertension, and long-term exposure to pulmonary heart disease. On the other hand, according to our study, the fact has been proven. Moreover, our findings indicate that siRNA-S100A9 interference and dorsomorphin may alleviate PM2.5-induced lung injury.

## Conclusion

In this study, we performed *in vivo *and* in vitro* PM2.5 exposure to confirm the key pathway of inducing lung epithelial cell damage. Our results demonstrated that PM2.5 exposure increased ROS levels and up-regulated the S100A9/AMPK signaling pathway to participate in the regulation of lung injury. This research raises awareness of the link between PM2.5 toxicity and lung tissue damage. Exploring new biomarkers to prevent and intervene in PM2.5-mediated lung tissue injury is also necessary. Further in-depth mechanistic research will be conducted in the future.

## Declaration

We have not used any AI tools or technologies to prepare this manuscript.
